# Characteristics of anal canal cancer in Japan

**DOI:** 10.1002/cam4.4631

**Published:** 2022-03-10

**Authors:** Kazutaka Yamada, Yasumitsu Saiki, Koji Komori, Akio Shiomi, Masashi Ueno, Masaaki Ito, Koya Hida, Seiichiro Yamamoto, Manabu Shiozawa, Soichiro Ishihara, Yukihide Kanemitsu, Hideki Ueno, Tatsuya Kinjo, Kotaro Maeda, Junichiro Kawamura, Fumihiko Fujita, Keiichi Takahashi, Tsunekazu Mizushima, Yasuhiro Shimada, Shin Sasaki, Eiji Sunami, Fumio Ishida, Keiji Hirata, Shinobu Ohnuma, Kimihiko Funahashi, Jun Watanabe, Yusuke Kinugasa, Shigeki Yamaguchi, Yojiro Hashiguchi, Masataka Ikeda, Takeshi Sudo, Yoshito Komatsu, Keiji Koda, Kazuhiro Sakamoto, Masazumi Okajima, Hideyuki Ishida, Yuichi Hisamatsu, Taiki Masuda, Shinichiro Mori, Kazuhito Minami, Seiji Hasegawa, Shungo Endo, Akinori Iwashita, Madoka Hamada, Yoichi Ajioka, Koichiro Usuku, Tokunori Ikeda, Kenichi Sugihara

**Affiliations:** ^1^ Department of Surgery Coloproctology Center Takano Hospital Kumamoto Japan; ^2^ Department of Gastroenterological Surgery Aichi Cancer Center Hospital Aichi Japan; ^3^ Division of Colon and Rectal Surgery Shizuoka Cancer Center Hospital Shizuoka Japan; ^4^ Department of Gastroenterological Surgery Cancer Institute Hospital of the Japanese Foundation for Cancer Research Tokyo Japan; ^5^ Department of Colorectal Surgery National Cancer Center Hospital East Chiba Japan; ^6^ Department of Surgery Kyoto University Hospital Kyoto Japan; ^7^ Department of Gastroenterological Surgery Tokai University School of Medicine Kanagawa Japan; ^8^ Department of Gastrointestinal Surgery Kanagawa Cancer Center Kanagawa Japan; ^9^ Department of Surgical Oncology The University of Tokyo Tokyo Japan; ^10^ Department of Colorectal Surgery National Cancer Center Hospital Tokyo Japan; ^11^ Department of Surgery National Defense Medical College Saitama Japan; ^12^ Department of Digestive and General Surgery, Graduate School of Medicine University of Ryukyus Okinawa Japan; ^13^ International Medical Center Fujita Health University Hospital Aichi Japan; ^14^ Department of Surgery Kindai University Faculty of Medicine Osaka Japan; ^15^ Department of Surgery Kurume University School of Medicine Fukuoka Japan; ^16^ Department of Surgery, Tokyo Metropolitan Cancer and Infectious Diseases Center Komagome Hospital Tokyo Japan; ^17^ Department of Gastroenterological Surgery, Graduate School of Medicine Osaka University Osaka Japan; ^18^ Department of Clinical Oncology Kochi Health Sciences Center Kochi Japan; ^19^ Department of Coloproctological Surgery Japanese Red Cross Medical Center Tokyo Japan; ^20^ Department of Surgery Kyorin University Faculty of Medicine Tokyo Japan; ^21^ Digestive Disease Center Showa University Northern Yokohama Hospital Kanagawa Japan; ^22^ Department of Surgery1, School of Medicine University of Occupational and Environmental Health Fukuoka Japan; ^23^ Department of Surgery Tohoku University Graduate School of Medicine Miyagi Japan; ^24^ Department of Gastroenterological Surgery Toho University Omori Medical Center Tokyo Japan; ^25^ Department of Surgery, Gastroenterological Center Yokohama City University Medical Center Kanagawa Japan; ^26^ Department of Gastrointestinal Surgery Tokyo Medical and Dental University Tokyo Japan; ^27^ Department of Gastroenterological Surgery Saitama Medical University International Medical Center Saitama Japan; ^28^ Department of Surgery Teikyo University School of Medicine Tokyo Japan; ^29^ Division of Lower Gastrointestinal Surgery, Department of Surgery Hyogo College of Medicine Hyogo Japan; ^30^ Department of Gastroenterological Surgery Yamagata Prefectural Central Hospital Yamagata Japan; ^31^ Department of Cancer Center Hokkaido University Hospital Hokkaido Japan; ^32^ Department of Surgery Teikyo University Chiba Medical Center Chiba Japan; ^33^ Department of Coloproctological Surgery Juntendo University Faculty of Medicine Tokyo Japan; ^34^ Department of Surgery Hiroshima City Hiroshima Citizens Hospital Hiroshima Japan; ^35^ Department of Digestive Tract and General Surgery, Saitama Medical Center Saitama Medical University Saitama Japan; ^36^ Department of Surgery and Science, Graduate School of Medical Sciences Kyushu University Fukuoka Japan; ^37^ Department of Surgery Tokyo Metropolitan Hiroo Hospital Tokyo Japan; ^38^ Department of Digestive Surgery, Breast and Thyroid Surgery, Graduate School of Medicine and Dental Sciences Kagoshima University Kagoshima Japan; ^39^ Department of Surgery Matsuyama Red Cross Hospital Ehime Japan; ^40^ Department of Surgery Saiseikai Yokohamashi Nanbu Hospital Kanagawa Japan; ^41^ Department of Coloproctology, Aizu Medical Center Fukushima Medical University Fukushima Japan; ^42^ Department of Pathology Fukuoka University Chikushi Hospital Fukuoka Japan; ^43^ Division of Gastrointestinal Surgery Kansai Medical University Hospital Osaka Japan; ^44^ Division of Molecular and Diagnostic Pathology Niigata University Graduate School of Medical and Dental Sciences Niigata Japan; ^45^ Department of Medical Information Sciences and Administration Planning Kumamoto University Hospital Kumamoto Japan; ^46^ Tokyo Medical and Dental University Tokyo Japan

**Keywords:** abdominoperineal resection, anal canal cancer, chemoradiotherapy, human papillomavirus, squamous cell carcinoma

## Abstract

Anal canal cancer (ACC) has been reported to be an uncommon cancer in Japan, as in the USA, Europe, and Australia. This retrospective multi‐institutional study was conducted to clarify the characteristics of ACC in Japan. First, the histological ACC type cases treated between 1991 and 2015 were collected. A detailed analysis of the characteristics of anal canal squamous cell carcinoma (SCC) cases was then conducted. The results of the histological types revealed that of the 1781 ACC cases, 435 cases (24.4%) including seven cases of adenosquamous cell carcinomas were SCC and 1260 cases (70.7%) were adenocarcinoma. However, the most common histological type reported in the USA, Europe, and Australia is SCC. Most ACC cases are adenocarcinomas and there is a low incidence of SCC in Japan which is different from the above‐mentioned countries. Moreover, we reclassified T4 into the following two groups based on tumor size: T4a (tumor diameter of 5 cm or less) and T4b (tumor diameter of more than 5 cm). The results of the TNM classification of SCC revealed that the hazard ratio (HR) to T1 of T2, T3, T4a, and T4b was 2.45, 2.28, 2.89, and 4.97, respectively. As T4b cases had a worse prognosis than T4a cases, we propose that T4 for anal canal SCC in Japan be subclassified into T4a and T4b.

## Graphical abstract

Most anal canal cancers in Japan are adenocarcinomas, the squamous cell carcinomas rate is low. In the T4 factor based on the depth of tumor invasion, there was a difference in prognosis depending on the tumor diameter and therefore we propose creating the subclassifications T4a and T4b for anal canal SCC in Japan.

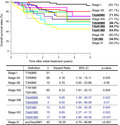

**List of abbreviations**
ACC: anal canal cancer
AJCC: American Joint Committee on Cancer
APR: abdominoperineal resection
CI: confidence interval
CRT: chemoradiotherapy
CT: chemotherapy
HIV: human immunodeficiency virus
HPV: human papillomavirus
HR: hazard ratio
JCCRC: Japanese Classification of Colorectal Appendiceal and Anal Carcinoma
JSCCR: Japanese society for cancer of the colon and rectum
OS: overall survival
RT: radiotherapy
SCC: squamous cell carcinoma
UICC: Union for International Cancer Control

## 1 INTRODUCTION

Anal canal cancer (ACC) is an uncommon tumor that represents 4% of all cancers in the lower gastrointestinal tract.^1^ In Japan, the new incidence rate of ACC is 0.10% of all cancers, the number of ACC deaths is 0.13% of all cancer deaths, and the 5‐year prevalence is 2.7 cases per 100,000 persons. However, in the United States, United Kingdom, and Australia, the new incidence rate is 0.37%, 0.37%, and 0.31% of all cancer types, respectively, which is similarly low to the values found in Japan. The number of ACC deaths is 0.22%, 0.32%, and 0.23% of all cancer deaths, respectively, and the 5‐year prevalence is 8.41, 8.19, and 8.13 cases per 100,000 persons, respectively.^2^

Previously, the anatomical definition of anal canal tumors in the literature varied, leading to confusion regarding diagnosis and treatment. At present, the definition of the anal canal has been revised in the TNM classification (8th edition), and the perianal skin (excluding the vulva) within 5 cm from the anal verge has been added along with the conventional surgical anal canal which corresponds to the tubular structure extending from the superior border of the puborectal sling to the anal verge,^3^, ^4^ and the treatment strategies for squamous cell carcinoma (SCC) and adenocarcinoma have been clarified. Although anal canal tumors arise from various different cells which constitute the anal canal, SCC is the most common, and the remaining comprise adenocarcinoma, melanoma, neuroendocrine tumor, sarcoma, gastrointestinal stromal tumor, and lymphoma.^5^, ^6^, ^7^, ^8^, ^9^ A growing body of evidence indicates that the oncogenic types of human papillomavirus (HPV), notably subtypes 16 and 18, are etiologically linked to develop SCC.^10^, ^11^ Although the treatment for ACC was mainly surgical resection by abdominoperineal resection (APR),^12^ chemoradiotherapy (CRT) has been proposed to be a useful treatment for SCC.^13^ The guidelines state that CRT is the main treatment for SCC of the anal canal. However, they also state that local resection is the primary treatment for some perianal SCC cases. The treatment strategy for adenocarcinoma and SCC in the anal canal is different: surgical resection for adenocarcinoma and CRT for SCC.^14^

The Japanese society for cancer of the colon and rectum (JSCCR) has provided the Japanese Classification of Colorectal, Appendiceal, and Anal Carcinoma (JCCRC)^15^ and treatment guidelines^16^ for colorectal cancer, and they are mainly for adenocarcinoma cases. However, anal canal SCC is classified according to the TNM classification (8th edition) and therefore, the classification of anal canal SCC in Japan needs to be examined.

As a result, the JSCCR conducted a retrospective multi‐institutional study aiming to clarify the characteristics of ACC including adenocarcinoma and SCC in Japan. In the present study, we describe the characteristics of anal canal SCC and propose a new classification of T factor for anal canal SCC in Japan.

## 2 PATIENTS AND METHODS

The patient records with malignant tumors of the anal canal who were treated between 1991 and 2015 were collected from 47 medical institutions in Japan. The anal canal is defined as the conventional surgical anal canal (P region) and the perianal skin (excluding the vulva) within 5 cm from the anal verge (E region) based on the TNM classification (8th edition).^3^, ^4^ Histological types of all cases were collected, and the 110 parameters of a questionnaire were used to investigate the characteristics of SCC in Japan: 23 for basic information (i.e., age, sex, and treatment methods) on each case, 38 for information on SCC (i.e., clinical and pathological findings), 35 for information on treatment (i.e., CRT regimens and surgical procedures), and 14 for information on prognosis (i.e., outcomes and recurrence). The basic information for female patients included HPV infection and the history of cervical cancer, vulvar cancer, and vaginal cancer, and the SCC information included the tumor diameter in T4 cases (tumors invading the surrounding organs regardless of size).

A total of 1781 patients were registered at the above‐mentioned 47 JSCCR affiliated institutions. Of these, 435 patients were SCC including seven adenosquamous cell carcinoma cases. There were 124 males and 311 females, with a mean age of 66.1 ± 12.0 years and prognosis analysis was performed for 295 anal canal SCC patients. 54 patients with unknown invasion depth, 105 with unknown maximum tumor size, 14 with unknown lymph node metastasis, nine with unknown survival time, and 10 with stage0 were excluded from the analysis.

T stage is based on the TNM classification (8th edition), but T4 was subclassified into two groups: T4a is defined as a T4 tumor with a diameter of 5 cm or less and T4b is as those with a diameter of more than 5 cm.

Consent to conduct the research was provided by the JSCCR ethical committee.

### 2.1 Statistical analysis

Survival curves were estimated using the Kaplan–Meier method and the stratified Cox proportional hazard model was used to estimate the hazard ratio (HR) and the 95% confidence interval (CI). The estimation using the Stratified Cox proportional hazard model was considered the following variables; age, sex, tumor location (P/E), treatment methods (CRT, RT, CT/surgery/untreated), N factor (N0/N1), and M factor (M0/M1). The variables that violated the proportional hazards assumption were used as stratification factors. All statistical analyses were performed using EZR (Saitama Medical Center, Jichi Medical University, Saitama, Japan, Version 1.36), which is a graphical user interface for R (the R Foundation for Statistical Computing, Version 3.4.1).^17^ More precisely, it is a modified version of R commander designed to add statistical functions frequently used in biostatistics.

## 3 RESULTS

### 3.1 Histological characteristic of ACC in Japan

A total of 1781 patients who were treated for ACC between January 1991 and December 2015 were registered from 47 JSCCR affiliated institutions. Of these, 428 patients (24.0%) were SCC, 7 (0.4%) were adenosquamous cell carcinoma, 1260 (70.7%) were adenocarcinoma and 16 were undifferentiated carcinoma (Table 1).

**TABLE 1:** Histological type of anal canal cancer in Japan (JSCCR questionnaire)

Histological type	No. of cases	%
Squamous cell carcinoma	428	24.0
1) Well differentiated	69	
2) Moderately differentiated	96	
3) Poorly differentiated	50	
4) Differentiation unknown	199	
5) Basaloid cell carcinoma	14	
Adenosquamous cell carcinoma	7	0.4
Adenocarcinoma	1260	70.7
1) Rectal type	778	
2) Extramucosal type	104	
3) Unknown	378	
Undifferentiated carcinoma	16	0.9
Other	70	3.9
Total	1781	

Abbreviation: JSCCR, Japanese society for cancer of the colon and rectum.

The clinicopathologic findings of 435 anal canal SCC cases including seven adenosquamous cell carcinoma cases are shown in Table 2. The mean age was 66.1 years, 124 patients (28.5%) were males and 311 patients (71.5%) were females. There were 407 tumors (93.6%) in the P region and 28 tumors (6.4%) in the E region. The most common macroscopic type was Type 2 (ulcerated type with clear margin),^15^ followed by Type 1 (polypoid type).^15^ Among the 435 patients with SCC or adenosquamous cell carcinoma, 35 patients and 256 patients were tested for HPV and for human immunodeficiency virus (HIV), respectively. The HPV‐ and HIV‐positive rate was 8.6% and 3.5%, respectively. There were no reports on the HPV antibody and HPV DNA test.

**TABLE 2:** Clinicopathologic findings and treatment methods for anal canal squamous cell carcinoma cases(435 cases: 1991–2015)

Baseline characteristics			TNM classification (8th edition)		
Mean age (SD) years	66.1	(12.0)	T factor		
Gender			TX	101	23.2%
Male	124	28.5%	Tis	10	2.3%
Female	311	71.5%	T1	73	16.8%
Tumor			T2	125	28.7%
Location			T3	33	7.6%
P^a^	407	93.6%	T4	93	21.4%
E^b^	28	6.4%	N factor		
Macroscopic type			NX	14	3.2%
Type 0	38	8.7%	N0	244	56.1%
Type 1	69	15.9%	N1a	152	34.9%
Type 2	157	36.1%	N1b	4	0.9%
Type 3	48	11.0%	N1c	21	4.8%
Type 4	2	0.5%	M factor		
Type 5	42	9.7%	M0	381	87.6%
Unknown	79	18.2%	M1	54	12.4%
HPV			Stage		
HPV test Yes: No	35: 400		Unknown	102	23.4%
HPV positive	3	8.6%	Stage 0	10	2.3%
HIV			Stage I	51	11.7%
HIV test Yes: No	256: 179		Stage IIA	68	15.6%
HIV positive	9	3.5%	Stage IIB	11	2.5%
Treatment method			Stage IIIA	60	13.8%
Chemoradiotherapy	259	59.5%	Stage IIIB	26	6.0%
Radiotherapy only	28	6.4%	Stage IIIC	53	12.2%
Chemotherapy only	5	1.1%	Stage IV	54	12.4%
Surgical treatment	132	30.3%			
Untreated	11	2.5%			

Abbreviations: HPV, human papillomavirus; HIV, human immunodeficiency virus.

^a^ P, surgical anal canal.

^b^ E, perianal skin (hair‐bearing skin within 5 cm of the anal verge excluding the vulva).

### 3.2 Treatment for anal canal SCC in Japan

CRT was done in 259 patients (59.5%), radiotherapy (RT) in 28 (6.4%), chemotherapy (CT) in 5 (1.1%), surgical treatment in 132 (30.3%), and no treatment in 11 (2.5%). Among surgical treatment, APR was performed in 79 patients, total pelvic exenteration in 8, local resection in 42, and other surgical procedures in 3. There has been a significant change in treatment over the past 25 years. CRT, RT, or CT were performed in 14.3% of the cases between 1991 and 2000, in 62.2% of the cases between 2001 and 2010, and in 84.3% of the cases between 2011 and 2015.

**FIGURE 1:**
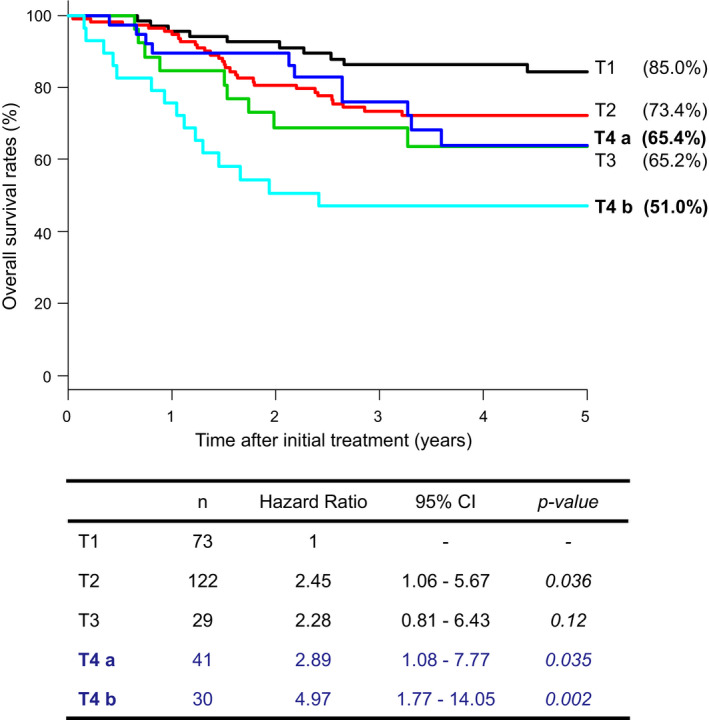
Overall survival rates and hazard ratios for each T factor case of anal canal squamous cell carcinoma, comparing the prognosis of T4a and T4b cases. Abbreviation: CI, confidence interval. T4a; T4 ≤5 cm, T4b; T4 >5 cm

The 5‐year overall survival (OS) rate for T1, T2, T3, T4a, and T4b cases was 85.0%, 73.4%, 65.2%, 65.4%, and 51.0%, respectively (Figure 1). The HR to T1 of T2, T3, T4a, and T4b cases was 2.45 (95% CI, 1.06–5.67; *p* = 0.036), 2.28 (95% CI, 0.81–6.43; *p* = 0.12), 2.89 (95% CI, 1.08–7.77; *p* = 0.035), and 4.97 (95% CI, 1.77–14.05; *p* = 0.002), respectively (Figure 1). An elevation in the HR induced by T factor progression was recognized. There was little difference in the 5‐year OS rate between T3 and T4a cases, but the HR showed a worse prognosis for T4a cases than for T3 cases. There was also no significant difference in HR between T4a and T4b cases, the prognosis worsened as the T factor progressed.

**FIGURE 2:**
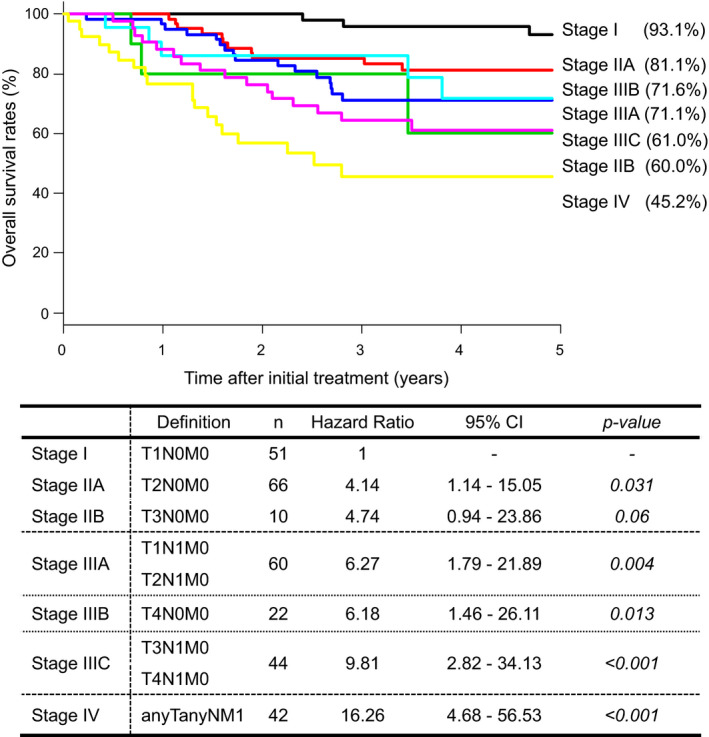
Treatment results based on the TNM classification (8th edition). Abbreviation: CI, confidence interval

**FIGURE 3:**
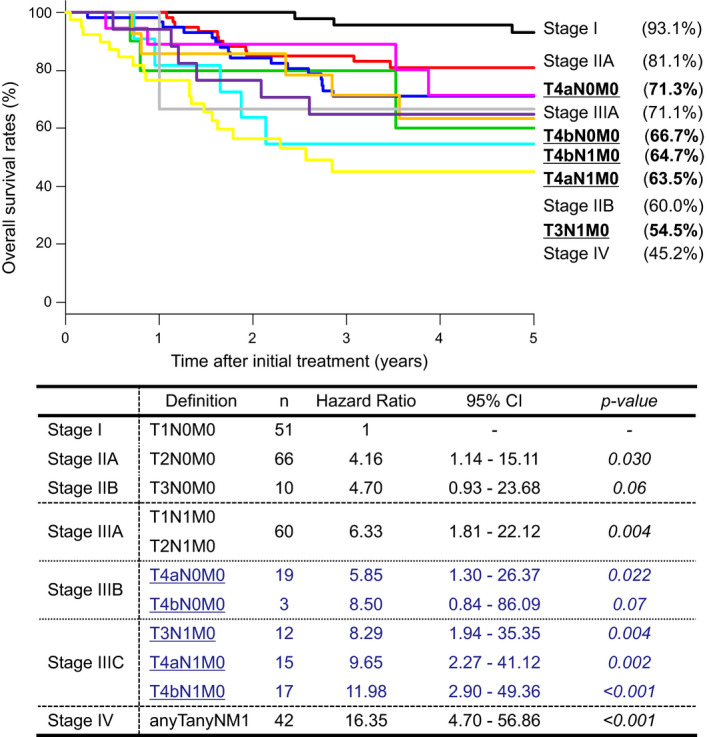
Review of the staging classification of anal canal squamous cell carcinoma incorporating the T4 subclassification. Abbreviation: CI, confidence interval. T4a; T4 ≤5 cm, T4b; T4 >5 cm

The 5‐year OS rate according to the TNM classification (8th edition) was 93.1% for stage I, 81.1% for stage IIA, 60.0% for stage IIB, 71.1% for stage IIIA, 71.6% for stage IIIB, 61.0% for stage IIIC, and 45.2% for stage IV. The HR was 1 for stageI, 4.14 (95% CI, 1.14–15.05; *p* = 0.031) for stage IIA, 4.74 (95% CI, 0.94–23.86; *p* = 0.06) for stage IIB, 6.27 (95% CI, 1.79–21.89; *p* = 0.004) for stage IIIA, 6.18 (95% CI, 1.46–26.11; *p* = 0.013) for stage IIIB, 9.81 (95% CI, 2.82–34.13; *p* < 0.001) for stageIIIC, and 16.26 (95% CI, 4.68–56.53; *p* < 0.001) for stage IV (Figure 2). When T4 is subclassified, new stage IIIB is also subclassified into T4aN0M0 and T4bN0M0, and new stage IIIC into T3N1M0, T4aN1M0, and T4bN1M0. The 5‐year OS rate according to the T4 subclassification was 71.3% for T4aN0M0, 66.7% for T4bN0M0, 54.5% for T3N1M0, 63.5% for T4aN1M0, and 64.7% for T4bN1M0. The HR according to the T4 subclassification was 1 for stage I, 5.85 (95% CI, 1.30–26.37; *p* = 0.022) for T4aN0M0, 8.50 (95% CI, 0.84–86.09; *p* = 0.07) for T4bN0M0, 8.29 (95% CI, 1.94–35.35; *p* = 0.004) for T3N1M0, 9.65 (95% CI, 2.27–41.12; *p* = 0.002) for T4aN1M0, and 11.98 (95% CI, 2.90–49.36; *p* < 0.001) for T4bN1M0 (Figure 3).

The 5‐year OS rate of 21 patients with extraregional lymph node metastasis (the inferior mesenteric trunk nodes, inferior mesenteric root nodes, or common iliac nodes^15^) was 58.9%, however, that of patients with other distant metastases was 31.9%.

## 4 DISCUSSION

In this study, the ratio of SCC including adenosquamous cell carcinoma to ACC was as low as 24.4%, and that of adenocarcinoma to ACC was as high as 70.7%. HPV and HIV infection was investigated as a characteristic of anal canal SCC cases, but only a few cases were tested for these infections. The treatment of SCC in Japan has changed over the past 25 years from surgical treatment to CRT, RT, and CT. Moreover, we reclassified T4 into the following two groups based on tumor size: T4a (tumor diameter of 5 cm or less) and T4b (tumor diameter of more than 5 cm). The results revealed that the T4b cases had a worse prognosis than T4a cases.

Anal cancer is an uncommon cancer, accounting for 0.43% of all malignancies, and the incidence rates in the United states have increased from 0.8 to 1.7 cases per 100,000 persons per year from 1975 to 2011.^18^ In the reports on ACC in the United Sttaes, the ratio of SCC was as high as 74.5%–84.6%, and that of adenocarcinoma was as low as 14.4%–18.8%.^5^, ^6^, ^7^ Similarly, in the United Kingdom, the ratio of SCC was at 68.3% and approximately 80%, and that of adenocarcinoma was at 21.2% and approximately 20%,^8^, ^9^ and the most common histologic type in Denmark^19^ and Australia^20^ is also SCC. On the other hand, in Japan, the ratio of SCC was low at 16.2%–24.4%, and that of adenocarcinoma was high at 66.8%–75.5%,^21^, ^22^ as with this study. These studies show that the ratio of SCC was very low in Japan, compared with the ACC in the United States, Europe, and Australia.

As risk factors for developing SCC of the anus, anal carcinoma is associated with HPV infection; a history of receptive anal intercourse or a sexually transmitted disease; a history of cervical, vulvar, or vaginal cancer; immunosuppression after solid organ transplantation or HIV infection; hematologic malignancies; certain autoimmune disorders; and smoking.^23^, ^24^, ^25^, ^26^, ^27^, ^28^, ^29^

Regarding the treatment of anal canal SCC, there has been a transition from surgical treatment to CRT,^12^, ^13^, ^14^ and there have also been changes in the reports of treatment methods. In addition, the National Comprehensive Cancer Network guidelines suggest that intensity‐modulated RT is preferred over 3D conformal RT in the treatment of anal canal SCC.^14^, ^30^ Notably, within each stage grouping, the 5‐year OS rates for ACC cases vary significantly according to the histologic type. At each stage, better survival rates for patients with SCC than that for those with nonsquamous tumors, including adenocarcinomas, have been reported.^31^

In the TNM classification, the description of ACC was presented from the Union for International Cancer Control (UICC) 4th edition (1987) and the American Joint Committee on Cancer (AJCC) 3rd edition (1988), and the definition of T factor had not changed until the current 8th edition (UICC, AJCC) .^3^, ^4^ Regarding the classification of T factor in digestive system tumors, esophageal cancer, gastric cancer, and small intestinal cancer as the upper gastrointestinal tract, and appendix cancer, colon cancer, rectal cancer, and ACC as the lower gastrointestinal tract, all cancers except ACC are classified based on the depth of tumor invasion. On the other hand, in liver cancer, pancreatic cancer, etc. as the hepatobiliary system, T1–T3 are mainly classified by tumor size, and T4 is classified based on the depth of tumor invasion. T4 in almost all cancers of the gastrointestinal tract is classified based on the depth of tumor invasion, but even in lacrimal gland carcinoma, T4 is subclassified by tumor invasion of the adjacent structure and tumor size.^3^, ^4^ In this study, we proposed a subclassification of the T4 for anal canal SCC by tumor size. The HR of T4aN0M0 is lower than the HR of T4bN0M0, and the HR of T4bN1M0 in stage IIIC is higher than the others. Therefore, it is considered appropriate to add T4aN0M0 to stage IIIA and to classify T4bN0M0, T3N1M0, and T4aN1M0 as stage IIIB, and to classify only T4bN1M0 as stage IIIC. The subclassification of T4 could be a valid prognostic factor, but this needs to be confirmed by future multicenter joint research.

On the other hand, the prognosis of stage IV in this study was relatively good, and the main reason for this was considered to be the good prognosis of patients with extraregional lymph node metastasis of anal canal SCC. The regional lymph nodes in ACC are the perirectal, internal iliac, external iliac, and inguinal lymph nodes; however, the inferior mesenteric trunk nodes, inferior mesenteric root nodes, and common iliac nodes are the extraregional lymph nodes. The AJCC has reported on a prognosis survey by stage for anal canal SCC,^4^, ^31^ but the current situation is that there are few reports from other institutions. Further studies will be needed to clarify whether it is appropriate that the inferior mesenteric trunk nodes, inferior mesenteric root nodes, and common iliac lymph nodes metastases are classified as stage IV.

Since this is a retrospective study, all information obtained was recorded using the criteria in place at the time of diagnosis and treatment. Ideally, it is best to collect cases prospectively by predetermining the examination criteria (i.e. tumor location criteria, tissue collection method, and pathological diagnosis criteria), but since ACC is an uncommon cancer, a long period of time is required to collect cases. ACC has been also historically subjected to various changes in staging and treatments, but there is little information on its prognosis, which needs to be investigated. ACC is an uncommon cancer, and this study showed that there are areas where SCC is common and areas where adenocarcinoma is common. Therefore, it is important to accumulate more data through studies from various perspectives.

In conclusion, we demonstrated that most ACC cases are adenocarcinomas and that there is a low incidence of SCC in Japan which is different from that in the United States, Europe, and Australia. Moreover, we propose subclassifying T4 for anal canal SCC in Japan into T4a and T4b based on tumor size.

## CONFLICT OF INTEREST

Kazutaka Yamada and the co‐authors have no conflicts of interest to declare.

## AUTHOR CONTRIBUTIONS

Kazutaka Yamada and Yasumitsu Saiki carried out concept; acquisition and performance of the analysis; drafting of the text, tables, and figures; responsibility for the overall content. Koji Komori, Akio Shiomi, Masashi Ueno, Masaaki Ito, Koya Hida, Seiichiro Yamamoto, Manabu Shiozawa, Soichiro Ishihara, Yukihide Kanemitsu, Hideki Ueno, Tatsuya Kinjo, Kotaro Maeda, Junichiro Kawamura, Fumihiko Fujita, Keiichi Takahashi, Tsunekazu Mizushima, Yasuhiro Shimada, Shin Sasaki, Eiji Sunami, Fumio Ishida, Keiji Hirata, Shinobu Ohnuma, Kimihiko Funahashi, Jun Watanabe, Yusuke Kinugasa, Shigeki Yamaguchi, Yojiro Hashiguchi, Masataka Ikeda, Takeshi Sudo, Yoshito Komatsu, Keiji Koda, Kazuhiro Sakamoto, Masazumi Okajima, Hideyuki Ishida, Yuichi Hisamatsu, Taiki Masuda, Shinichiro Mori, Kazuhito Minami, Seiji Hasegawa, Shungo Endo, Akinori Iwashita, Madoka Hamada, and Yoichi Ajioka were involved in acquisition and interpretation of data. Koichiro Usuku and Tokunori Ikeda performed the statistical analysis. Kenichi Sugihara was involved in the drafting of the article. All authors reviewed the final document and approved it for publication.

## ETHICS STATEMENT

This study was approved by the Institutional Review Board of the JSCCR. This study was also approved by the Hospital Review Board of each hospital. Informed consent was obtained using an “opt‐out” method under the approval of the ethics committee. The study was performed in accordance with the Declaration of Helsinki.

## Data Availability

All data generated or analyzed during this study are included in this published article.
